# Heterotopic connectivity of callosal dysgenesis in mice and humans

**DOI:** 10.3389/fnins.2023.1191859

**Published:** 2023-05-18

**Authors:** Diego Szczupak, Roberto Lent, Fernanda Tovar-Moll, Afonso C. Silva

**Affiliations:** ^1^Department of Neurobiology, University of Pittsburgh Brain Institute, University of Pittsburgh, Pittsburgh, PA, United States; ^2^Institute of Biomedical Sciences, Federal University of Rio de Janeiro (UFRJ), Rio de Janeiro, Brazil; ^3^D’Or Institute Research and Education (IDOR), Rio de Janeiro, Brazil

**Keywords:** corpus callosum, heterotopicity, human, mouse, structural brain connectivity, tractography, callosal dysgenesis

## Abstract

The corpus callosum (CC), the largest brain commissure and the primary white matter pathway for interhemispheric cortical connectivity, was traditionally viewed as a predominantly homotopic structure, connecting mirror areas of the cortex. However, new studies verified that most callosal commissural fibers are heterotopic. Recently, we reported that ~75% of the callosal connections in the brains of mice, marmosets, and humans are heterotopic, having an essential role in determining the global properties of brain networks. In the present study, we leveraged high-resolution diffusion-weighted imaging and graph network modeling to investigate the relationship between heterotopic and homotopic callosal fibers in human subjects and in a spontaneous mouse model of Corpus Callosum Dysgenesis (CCD), a congenital developmental CC malformation that leads to widespread whole-brain reorganization. Our results show that the CCD brain is more heterotopic than the normotypical brain, with both mouse and human CCD subjects displaying highly variable heterotopicity maps. CCD mice have a clear heterotopicity cluster in the anterior CC, while hypoplasic humans have strongly variable patterns. Graph network-based connectivity profile showed a direct impact of heterotopic connections on CCD brains altering several network-based statistics. Our collective results show that CCD directly alters heterotopic connections and brain connectivity.

## Introduction

Corpus Callosum Dysgenesis (CCD) is a congenital developmental malformation of the corpus callosum (CC; Probst, 1901; Brown and Paul, 2019) often associated with several comorbidities (Paul, 2011). Callosal malformations vary extensively in their presentation, from hypoplasic (reduced midsagittal CC area) and partial (usually with a rostral CC remnant in humans and caudal in mice) formations to complete agenesis (Tovar-Moll et al., 2006). Regardless of the degree of callosal malformations, reorganization of the CCD brain can generate new aberrant white matter fiber bundles (Probst, 1901; Tovar-Moll et al., 2006; Paul et al., 2007; Kasprian et al., 2013; Severino et al., 2017; Edwards et al., 2020; Szczupak et al., 2020a), leading to widespread structural (Owen et al., 2013b; Jakab et al., 2015; Siffredi et al., 2020; Szczupak et al., 2021b), and functional (Owen et al., 2013a; Tovar-Moll et al., 2014; Lazarev et al., 2016; Monteiro et al., 2019; Szczupak et al., 2020b, 2021b) changes affecting the entire brain. For example, hypoplasic CCD subjects and mouse models have an atypical structural brain connectivity profile that is substantially different from that of healthy subjects (Owen et al., 2013b; Edwards et al., 2020; Szczupak et al., 2020a, 2021b; Siffredi et al., 2021).

Until recently, the CC was traditionally viewed as a predominantly homotopic structure, connecting mirror areas of the cortex (Zhou et al., 2013; Fenlon and Richards, 2015; Shen et al., 2015; Roland et al., 2017; Suárez et al., 2018; Mancuso et al., 2019; Loomba et al., 2021). However, the latest studies verified that most callosal commissural fibers are heterotopic (Swanson et al., 2017; Szczupak et al., 2022), raising the possibility that the CC participates in a much broader integration of global brain connectivity than previously thought. We reported that ~75% of the callosal connections in the brains of mice, marmosets, and humans are heterotopic, having an essential role in determining the global properties of brain networks (Szczupak et al., 2022). However, the relative proportion between homotopic and heterotopic interhemispheric connections in the CCD brain remains unexplored. In the present study, we used high-resolution diffusion-weighted imaging and graph network modeling to investigate the balance of heterotopic and homotopic callosal connections in human subjects with callosal hypoplasia and a spontaneous murine model of CCD, the Balb/c mouse strain.

## Methods

### Diffusion-weighted MRI acquisitions

#### C57bl6 mice

The DWI data of 17 C57BL6/J mice (10 males, 7 females) were obtained in a 16.4 T magnet, as described by Liu et al. (2016). Briefly, the animals were sacrificed, and their brains were perfused with PFA and treated with gadolinium (Liu et al., 2016). The fixed brains were then imaged with a 100 μm isotropic high-resolution using a standard spin-echo sequence with the following parameters: TR = 400 ms, TE = 20 ms, δ/Δ = 2.5/12 ms, FOV = 18.99 × 11.16 × 8 mm, matrix size = 190 × 112 × 80, bandwidth = 50 kHz, 30 diffusion-encoding directions with b-value = 5,000 s/mm^2^, and 2 b0 images acquired without diffusion-weighting (b = 0 s/mm^2^).

#### Balb/C mice

The DWI data of 8 Balb/c mice (4 males, 4 females) were obtained in a 14 T vertical bore small animal MRI system equipped with a micro 2.5 gradient system and a 15 mm coil, as described in Szczupak et al. (2020a). Briefly, diffusion-weighted 3-D spin-echo EPI images were acquired using the following parameters: TR/TE = 450/21 ms, δ/Δ = 3/7.5 ms, FOV = 12.80 × 10.24 × 6.40 mm^3^, matrix size = 160 × 128 × 80 yielding an isotropic resolution of 80 μm, number of averages = 2, bandwidth = 300 kHz, 232 diffusion-weighting directions split in three shells of 39, 77, and 116 directions, b-value = 1,500, 3,000, and 6,000 s/mm^2^ with 4 b0 images. To increase comparability between sequences and strains, we only used 30 directions of the b6000 shell from this acquisition in the data processing.

### Humans

#### Controls

The DWI data of 51 human subjects (21 males and 20 females) were obtained at 3 T from the HCP 100 unrelated subjects database (Van Essen et al., 2013). The DWI data used standard echo-planar imaging (EPI) with the HCP standard protocol parameters: 200 diffusion-encoding directions with two shells, b-values of 1,500 s/mm^2^ and 3,000 s/mm^2^, 1.5 mm isotropic resolution acquired in two different phase encoding directions to minimize drop-out signal and EPI distortion.

#### CCD

CCD patient data were previously published in Szczupak et al. (2021b). Briefly, we recruited 5 CCD patients presenting callosal hypoplasia. An experienced neurologist performed the diagnosis after a multimodal MRI examination. The Ethics Committee of our institution approved all procedures, and we obtained written informed consent from the patients or their parents. Image data were acquired following the Human Connectome Project (HCP) criteria for 3 T machines to ensure comparability with control HCP data.

### Structural connectomes

#### Callosal and whole-brain tractograms

DWI images were corrected for eddy-currents (Andersson and Sotiropoulos, 2016) and denoised (Tournier et al., 2012). Furthermore, we estimated a response function using the Dholander algorithm (Dhollander et al., 2019) and calculated the fiber orientation distribution (FOD) in MRtrix (Tournier et al., 2007). We then calculated the callosal tractogram (“callosogram”) by seeding the entire brain and selecting 1 M streamlines with the midline CC as an inclusion ROI to assure enough coverage and representativity of the tractogram. The whole-brain tractogram, also containing 1 M streamlines, was calculated without using the midline CC as an inclusion ROI.

#### Connectome adjacency matrices

For both mice and human data, we registered the QBI mice (Jiang and Johnson, 2011) and AAL 116 ROIs human (Tzourio-Mazoyer et al., 2002) atlases to their respective FODs and used the command tck2connectome (Tournier et al., 2019) to calculate the connectome adjacency matrices, following a previously established pipeline (Szczupak et al., 2022).

#### Heterotopicity index maps

Heterotopicity index maps were calculated using a previously published pipeline (Szczupak et al., 2022). Briefly, the heterotopicity index was defined as the number of heterotopic interhemispheric (callosal) connections (streamlines) divided by the total number of interhemispheric connections of the same cortical region. The heterotopicity index was calculated for every cortical area of every subject and 3D-rendered in Mango. This approach allows the direct comparison of heterotopicity index maps across species and different DWI protocols, as it normalizes the heterotopic connections by the interhemispheric connectivity at the individual level.

#### Heterotopicity callosal maps

Callosal heterotopicity maps were calculated using a previously published pipeline (Szczupak et al., 2022). Tractography of every cortical region to all other contralateral cortical regions was performed, a tract density image was generated, masked to the midline corpus callosum, normalized, and a voxel-to-voxel operation was performed to calculate the heterotopicity index for each callosal voxel.

### Network-based-statistics

We used the GRETNA toolbox (Wang et al., 2015) automated software to calculate the NBS of mice and humans based on diffusion-weighted structural connectivity. We chose to evaluate the global network properties of Efficiency, Small Worldliness, Hierarchy, and Assortativity to understand how heterotopic connections influence the whole-brain network. Efficiency is the number of paths connecting two nodes and relates to efficiency and the network’s redundancy. Small Worldliness is how the network approaches a pure small world motif (many short-range connections and few long-range integrative connections), usually associated with high communication efficiency and information transfer reliability. On the other hand, Hierarchy comprises classifying individual nodes (e.g., MRI atlas ROIs) according to each node’s degree (number of connections). Finally, Assortativity defines if these nodes communicate with nodes of a similar class, relating to the network’s pattern and type of connectivity (Sporns et al., 2005).

### Non-heterotopic network generation

We calculated the non-heterotopic (NH) network by subtracting the heterotopic connections from the whole-brain connectome full network (FN). This way, we could compare the whole-brain connectome with and without the heterotopic connections. The delta NBS values were obtained by subtracting the NH values from each subject’s FN values, leaving only the heterotopic contribution to the NBS. We evaluated the Network-Based-Statistics with a pairwise parametric t-test and the deltas with an unpaired parametric *t*-test using GraphPad 7.0 Software (GraphPad Inc.).

## Results

### Heterotopicity index maps

#### Mice

**Figure 1:**
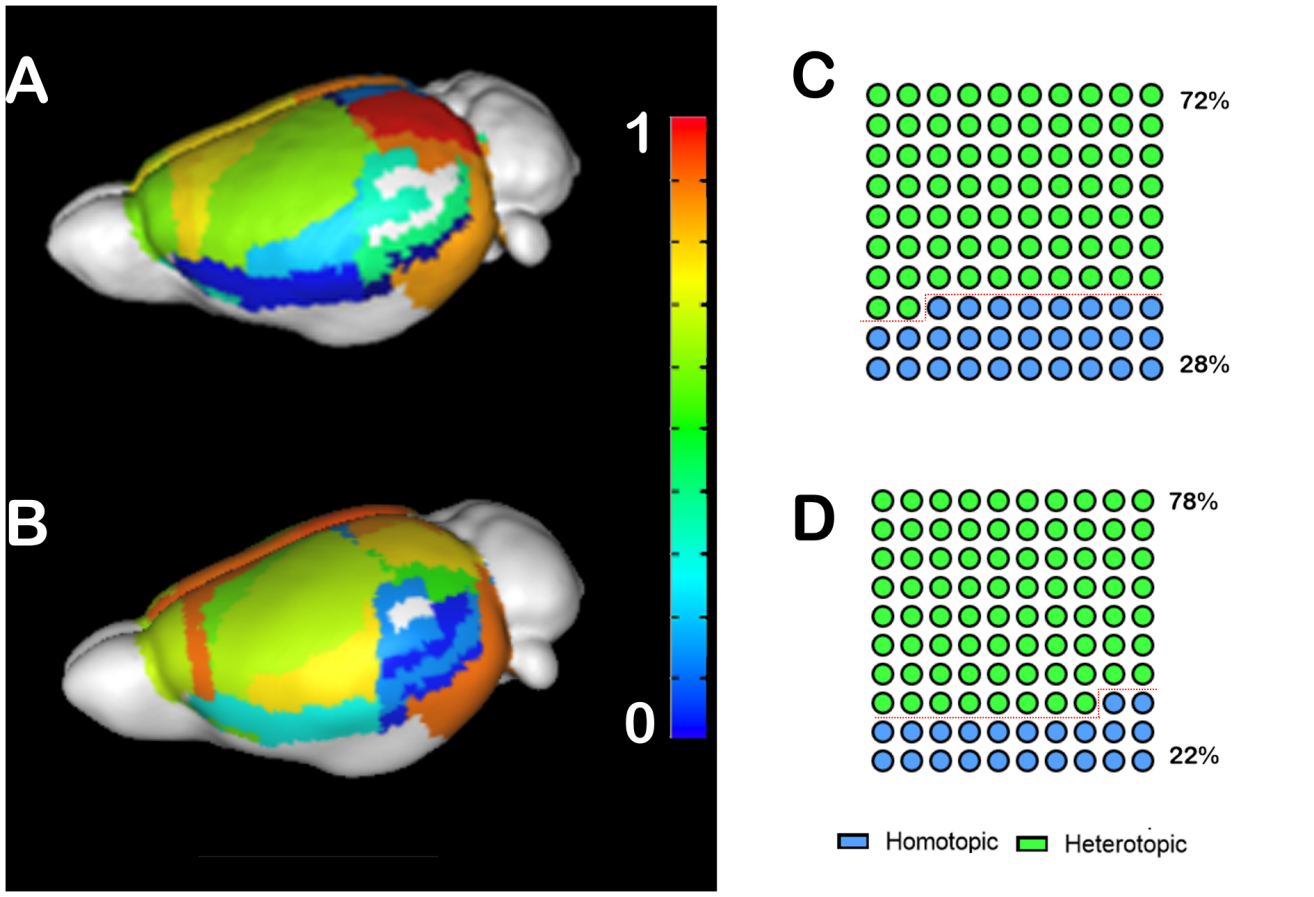
Heterotopicity maps of the mouse cortex in C57bl6/J and Balb/c mice. DWI callosal tractography-based heterotopicity populational average map of the C57bl6/J **(A)** and the Balb/c CCD mouse model **(B)** mice cortex. The color bar represents the heterotopicity index scale, with cool colors showing homotopic areas and hot colors showing heterotopic areas. Quantification of the interhemispheric cortical connectivity shows that 72% of the C57bl6/J **(C)** and 78% of the Balb/c callosal connections are heterotopic **(D)**, revealing a clear impact of callosal dysgenesis on the heterotopic connectivity.

To investigate the impact of CCD on interhemispheric heterotopic connections, we generated callosograms (CC tractograms) and plotted average populational cortical heterotopicity index maps for the C57bl6/J mice (Figure 1A) and the CCD mouse model Balb/c (Figure 1B). These maps show that the primary somatosensory cortex has a similar heterotopicity index in both strains, but the medial regions have a much higher heterotopicity index in the Balb/c. This increase in heterotopicity can be clearly seen in the global heterotopicity quantification, where 72% of the callosal connections in C57bl6/J are heterotopic (Figure 1C) vs. 78% in Balb/c mice (Figure 1D).

#### Humans

**Figure 2:**
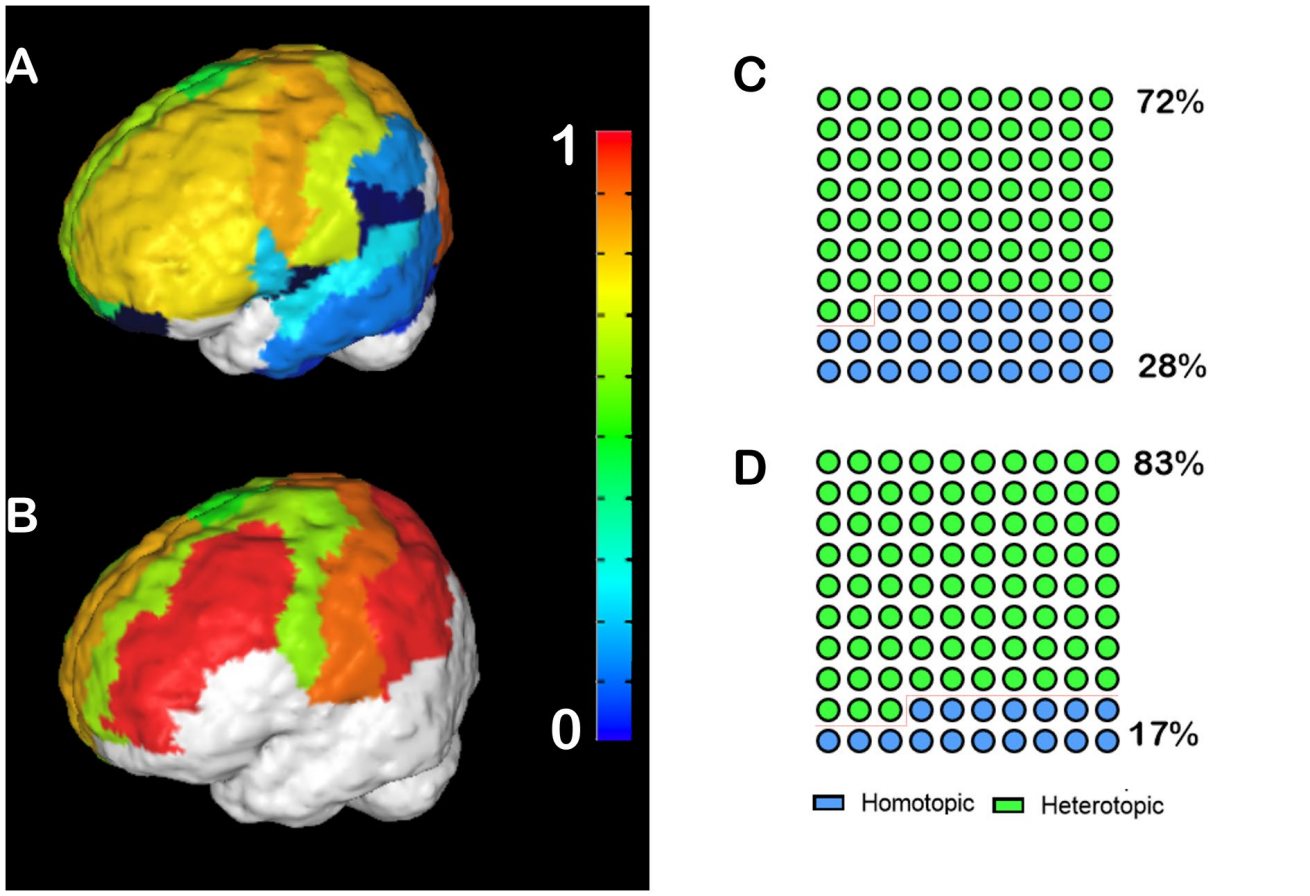
Heterotopicity map of healthy human controls and CCD patients. DWI callosal tractography-based heterotopicity populational average maps of typical subjects **(A)** and CCD hypoplasic subjects **(B)**. The color bar represents the heterotopicity index scale, with cool colors showing homotopic areas and hot colors showing heterotopic areas. Quantification of the interhemispheric cortical connectivity shows that 72% of typical subjects **(C)** and 83% of hypoplasic connections are heterotopic **(D)**, revealing a clear impact of callosal dysgenesis on the heterotopic connectivity.

We performed the same analysis for human CCD subjects, in which we noticed a different pattern. Control human patients have high heterotopicity indexes in the frontal, parietal, and posterior occipital cortices, and low heterotopicity in the lateral occipital cortex and the temporal lobe (Figure 2A). Meanwhile, hypoplasic subjects presented several clusters of high heterotopicity and only a few areas of low heterotopicity (Figure 2B). Since many brain regions in CCD subjects were unable to cross the midline, these areas are not congruent with the control subjects’ heterotopicity, indicating a whole-brain reorganization. Like CCD mice, 83% of the callosal connections in hypoplasic humans subjects are heterotopic (Figure 2D), compared to 78% in control subjects (Figure 2C).

#### Individual anatomical variance

**Figure 3:**
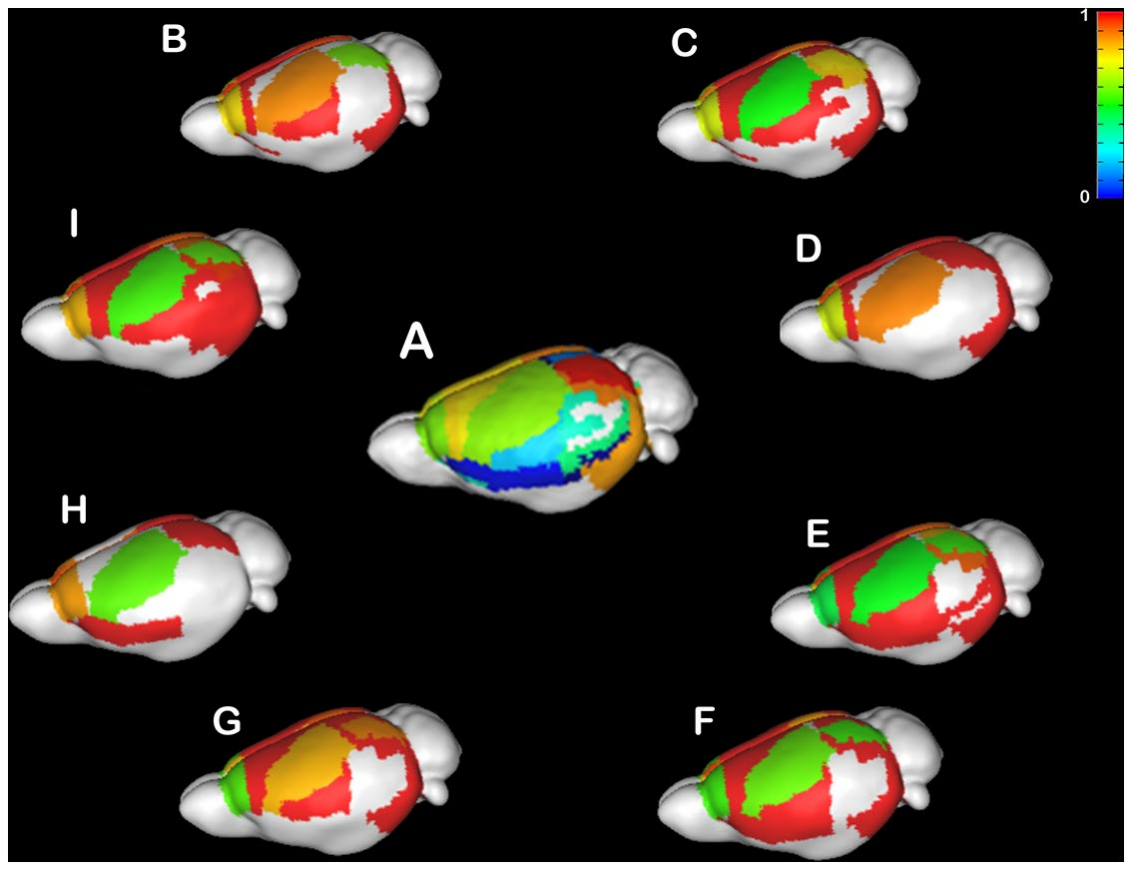
Individual Heterotopicity map of Balb/c mice. DWI callosal tractography-based heterotopicity maps of the populational average C57bl6/J mouse **(A)** and each Balb/c CCD mouse model **(B–I)**. The color bar represents the heterotopicity index scale, with cool colors showing homotopic areas and hot colors showing heterotopic areas. The individual maps reveal a large individual variance of each animal relative to the average map of C57bl6/J.

**Figure 4:**
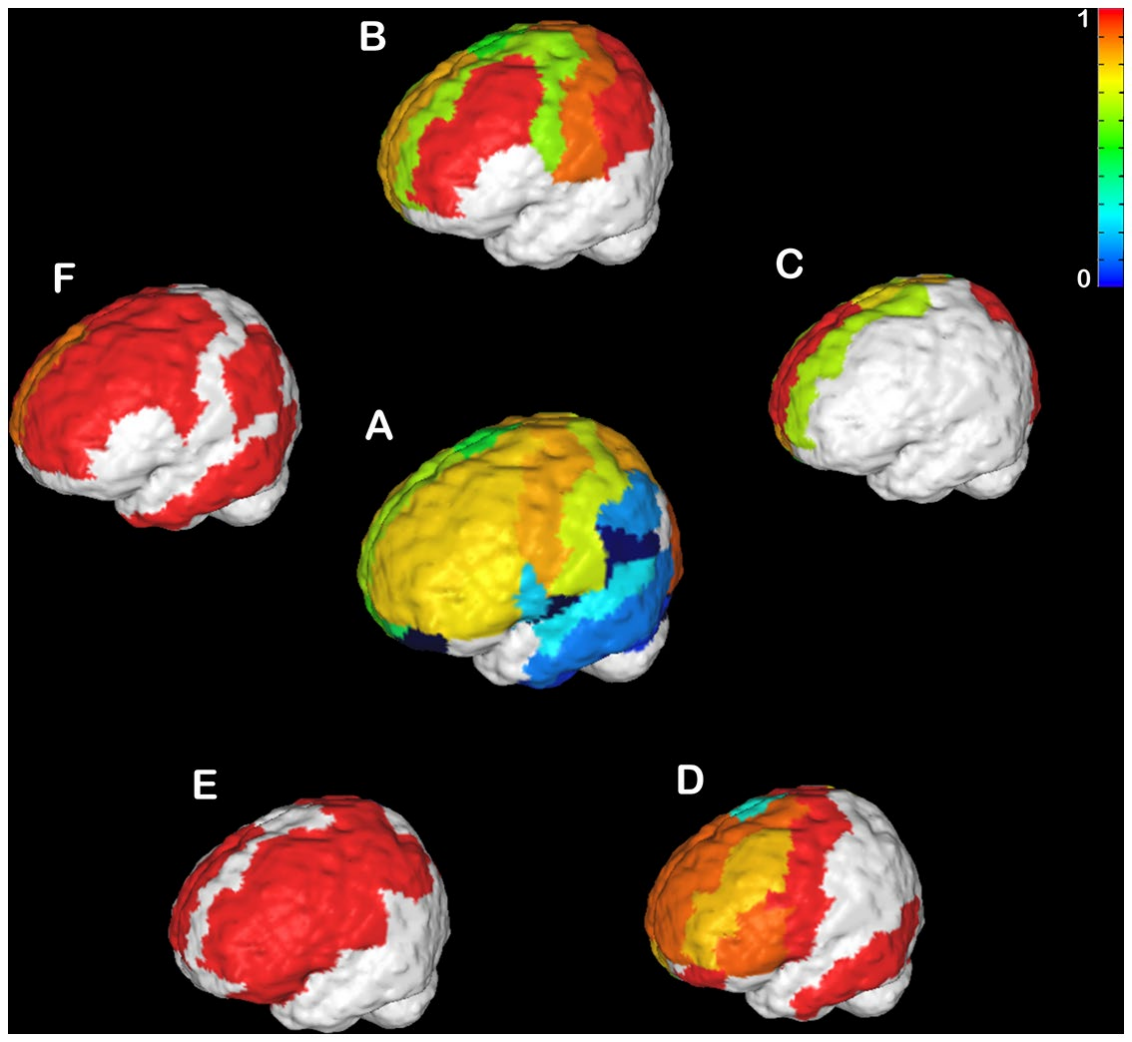
Individual Heterotopicity map of hypoplasic CCD subjects. DWI callosal tractography-based heterotopicity maps of the populational average typical subject **(A)** and each hypoplasic CCD subject **(B–D)**. The color bar represents the scale of heterotopicity, with cool colors showing homotopic areas and hot colors showing heterotopic areas. The individual maps reveal a large individual variance of each subject relative to the average map of typical individuals.

CCD is an incredibly variable condition in humans (Tovar-Moll et al., 2006; Paul, 2011; Owen et al., 2013a,b; Jakab et al., 2015; Siffredi et al., 2019; Szczupak et al., 2021b) and mice (Wahlsten et al., 1992, 2003; Edwards et al., 2020; Szczupak et al., 2020a). Therefore, it is crucial to understand if the heterotopicity in CCD brains is also variable. Here, we investigated the variability in heterotopicity across subjects in mice (Figure 3) and humans (Figure 4).

It is noticeable that the Balb/c mice have a wide range of heterotopicity indexes in different cortical regions (Figures 3B–I) relative to C57bl6/J (Figure 3A). However, a pattern is discernible in that the somatosensory cortex has lower heterotopicity indexes than surrounding medial and parietal areas and the temporal lobe has barely any connectivity.

An even more variable pattern is found in hypoplasic humans (Figures 4B–F). No clear pattern could be observed across all CCD patients. Instead, there were several clusters of very high heterotopicity, few areas with low heterotopicity, and many areas that are not connected. This high variance across subjects is likely related to their different etiology and CC malformations, which affect brain connectivity in heterogenous ways (Owen et al., 2013b; Jakab et al., 2015; Siffredi et al., 2021; Szczupak et al., 2021b).

### Heterotopicity callosal map

**Figure 5:**
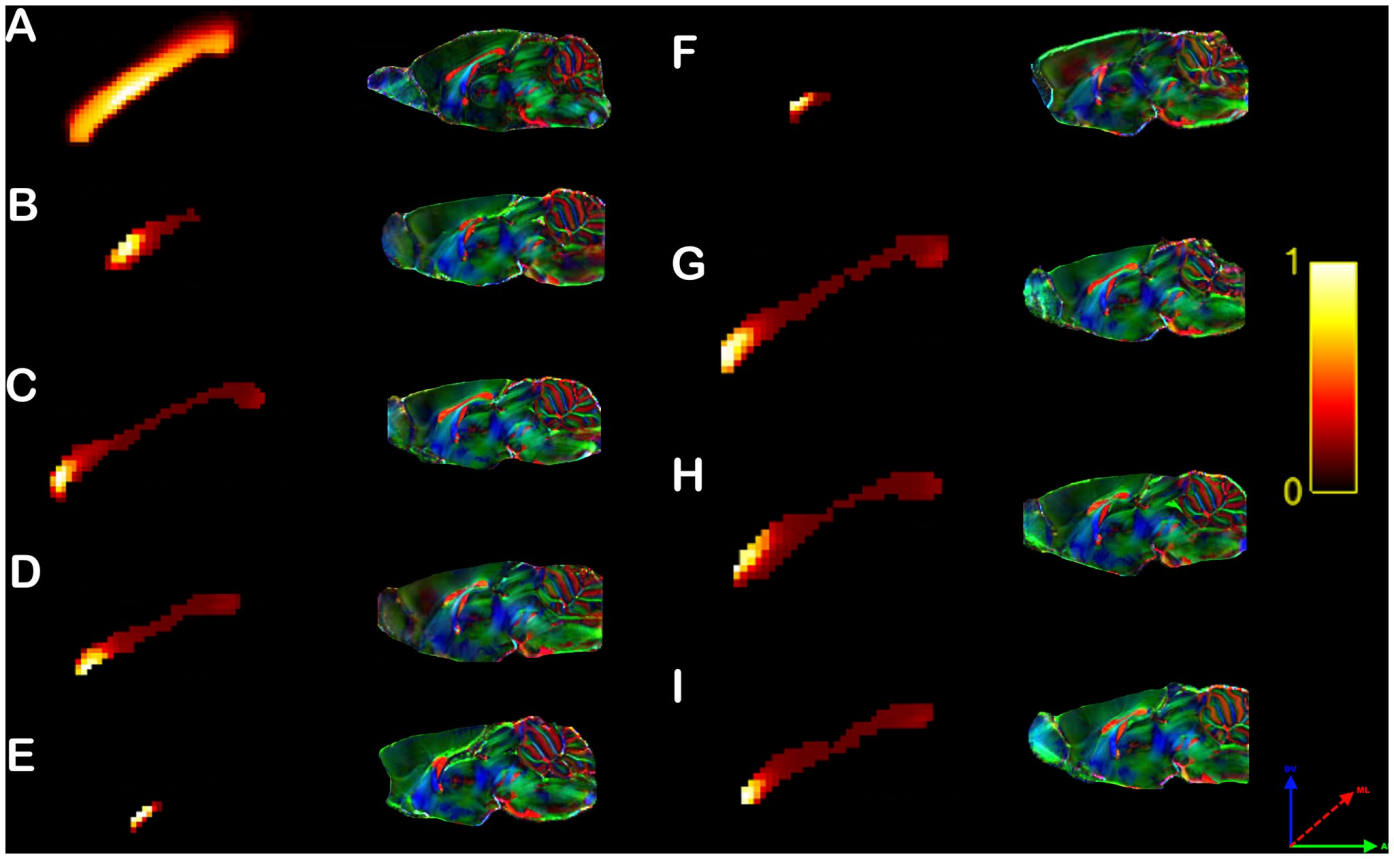
Rodent callosal heterotopicity map. Voxel-based maps of the corpus callosum heterotopicity and color-coded FOD maps of the C57bl6/J populational average **(A)** and all individual Balb/c mice **(B–I)**. Heterotopicity callosal maps reveal a predominantly rostral connectivity hub in Balb/c mice relative to C57bl6/J normotypical mice. The red axis represents mediolateral (ML) diffusion, the green axis anteroposterior (AP) diffusion, and the blue axis dorsoventral (DV) diffusion.

In order to understand the anatomical placement of the heterotopic connections within the CC, we mapped the interhemispheric connections in each midline callosal voxel and calculated their heterotopicity indexes for each subject. In C57bl6/J mice, the heterotopic connections are distributed along the anteroposterior axis but concentrated in the center of the CC (Figure 5A), while in the CCD Balb/c mouse model, there is clearly a cluster of high heterotopicity in the genu of the CC (Figures 5C,D,G–I) or CC remnants (Figures 5B,E,F).

**Figure 6:**
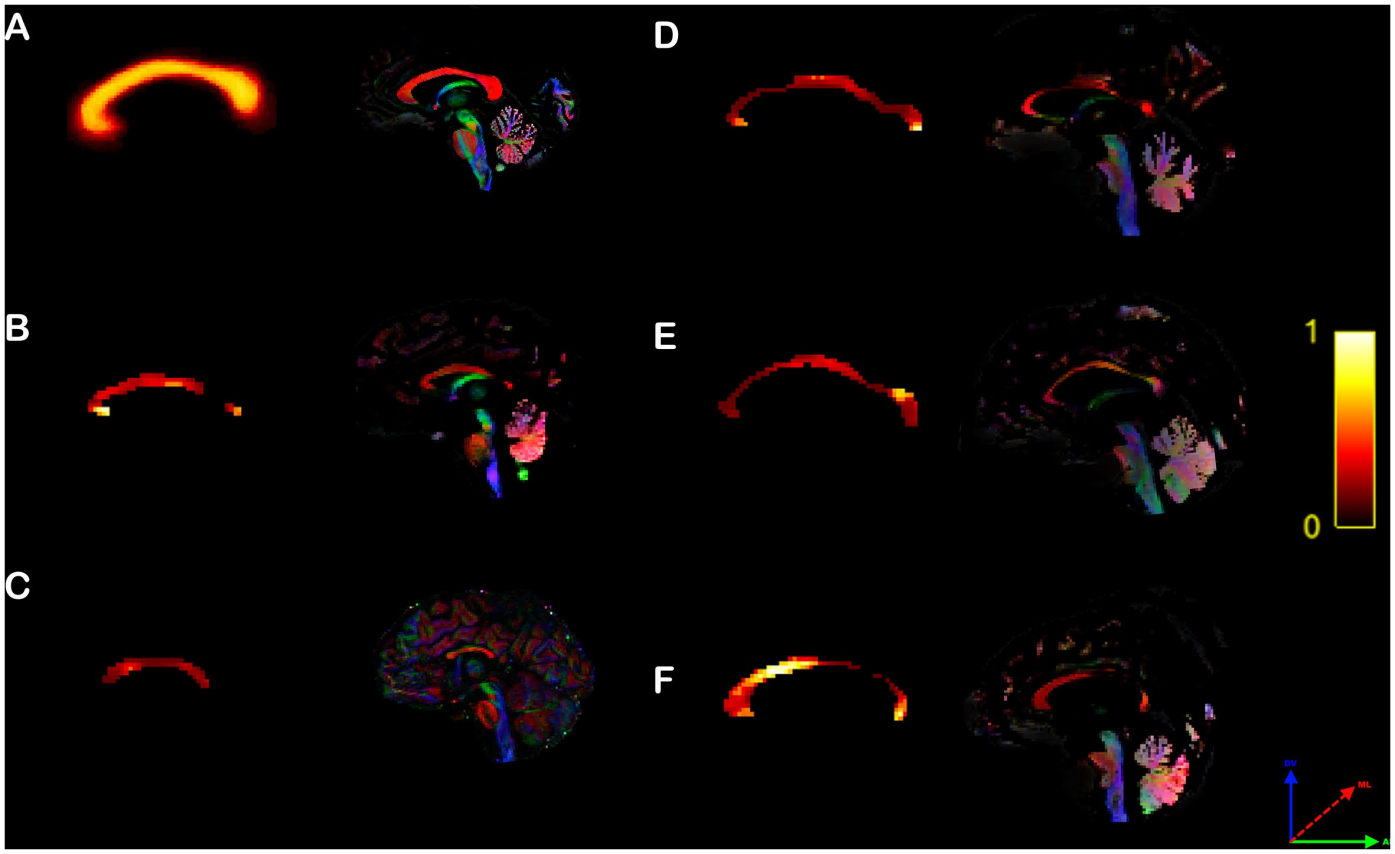
Human callosal heterotopicity map. Voxel-based maps of the corpus callosum heterotopicity and its color-coded FOD map of the typical subject populational average **(A)** and all individual hypoplasic subjects **(B–F)**. Heterotopicity callosal map reveals a wide variance in hypoplasic subjects, with focal clusters of high heterotopicity interspersed along the anteroposterior axis of the CC, relative to typical subjects, which showed a homogeneously distributed core of high heterotopicity surrounded by homotopic connections along the CC periphery. The red axis represents mediolateral (ML) diffusion, the green axis anteroposterior (AP) diffusion, and the blue axis dorsoventral (DV) diffusion.

Control human subjects displayed heterotopic callosal connections that were distributed along the core of the CC, and surrounded by homotopic connections dispersed along its periphery (Figure 6A), as previously published (Szczupak et al., 2022). However, similar to their heterotopicity of individual cortical maps, hypoplasic CCD subjects showed variable heterotopicity topographies with specific focal clusters of high heterotopicity (Figures 6B–F).

### Importance of heterotopic connections to network-based-statistics

**Figure 7:**
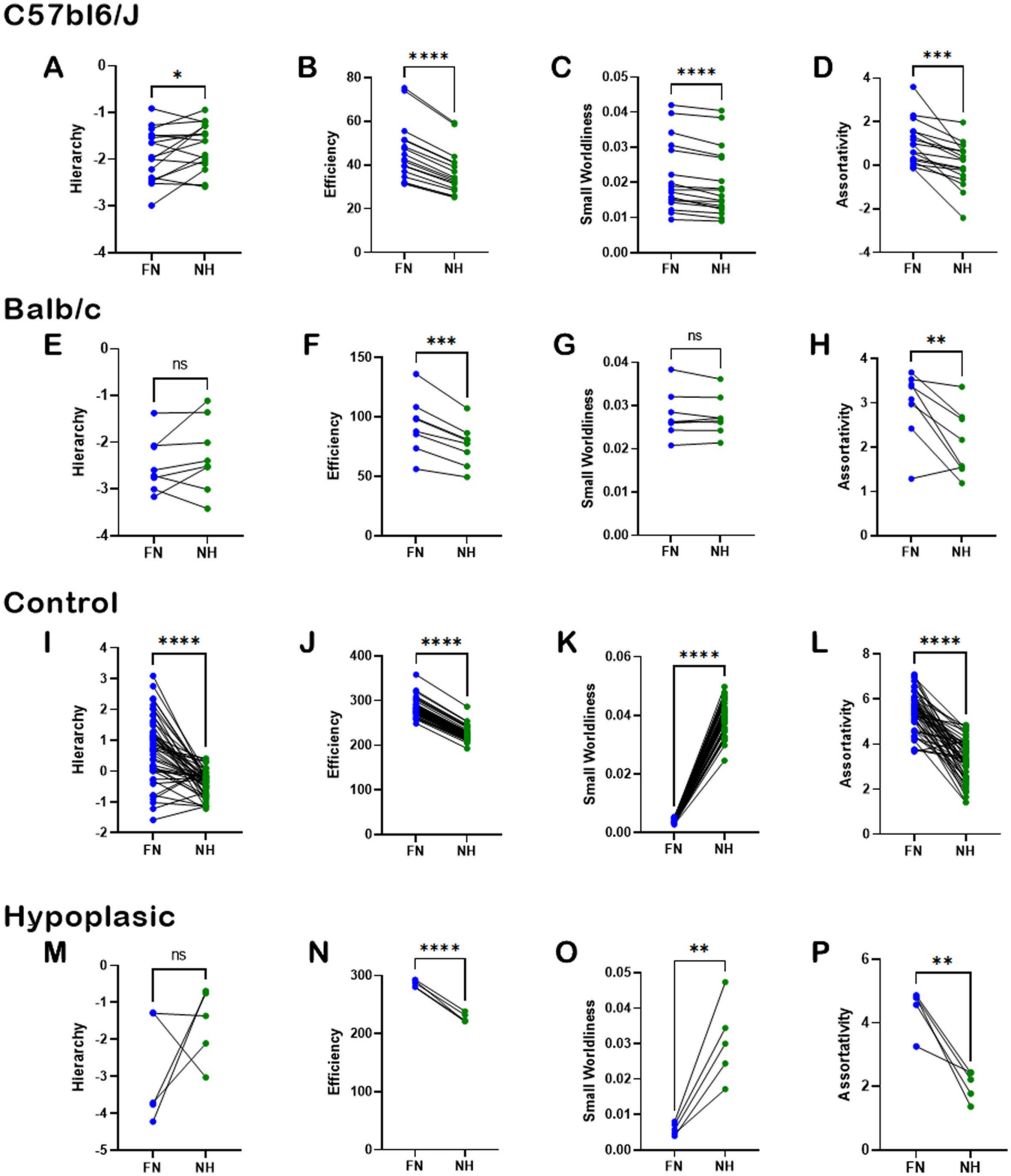
Importance of heterotopic connections to network-based-statistics. Pairwise analysis of the impact of heterotopic connections on whole-brain network-based-statistics in C5bl6/J control mice **(A–D)**, Balb/c CCD mice **(E–H)**, healthy human subjects **(I–L)**, and hypoplasic CCD patients **(M–P)**. The network properties of Hierarchy, Efficiency, Small Worldliness, and Assortativity were computed for the full network (FN) and after removing the heterotopic connections (NH). There was a clear impact of removing the heterotopic connections to most network properties, revealing the importance of such connections to brain function. Ns, not significant; ^*^p < 0.05, ^**^p < 0.01, ^***^p < 0.001, and ^****^p < 0.0001; two-tailed paired *t*-tests.

To understand the importance of heterotopic connections to the overall brain function, we used graph theory and network-based statistics to compute the properties of Hierarchy, Efficiency, Small Worldliness, and Assortativity under two conditions. First, the NBS properties were calculated using the full brain network (FN). Then, we removed the heterotopic connections, and assessed the NBS properties for the remaining non-heterotopic network (NH, comprising intrahemispheric and homotopic connections). The data for normal brains and CCD individuals are shown in Figure 7. In the C57bl6/J strain, heterotopic connections contributed significantly to all tested NBS properties (Figures 7A–D). However, removing heterotopic connections in the CCD mouse Balb/c impacted only Efficiency and Assortativity (Figures 7F,H), but not Hierarchy and Assortativity (Figures 7E,G). These results indicate that in the Balb/c mouse brain, the heterotopic streamlines connect different regions and node classes than in the C57bl6/J brain.

Similar results were found in humans. Removal of heterotopic connections severely impacted all NBS properties in healthy subjects (Figures 7I–L), and all but Hierarchy in hypoplasic CCD patients (Figures 7M–P). As Hierarchy presented the highest variance across human subjects, the lack of significance between FN and NH is likely due to the small sample size associated with the heterogeneity of the CCD condition.

### Relevance of heterotopic connections to CCD

**Figure 8:**
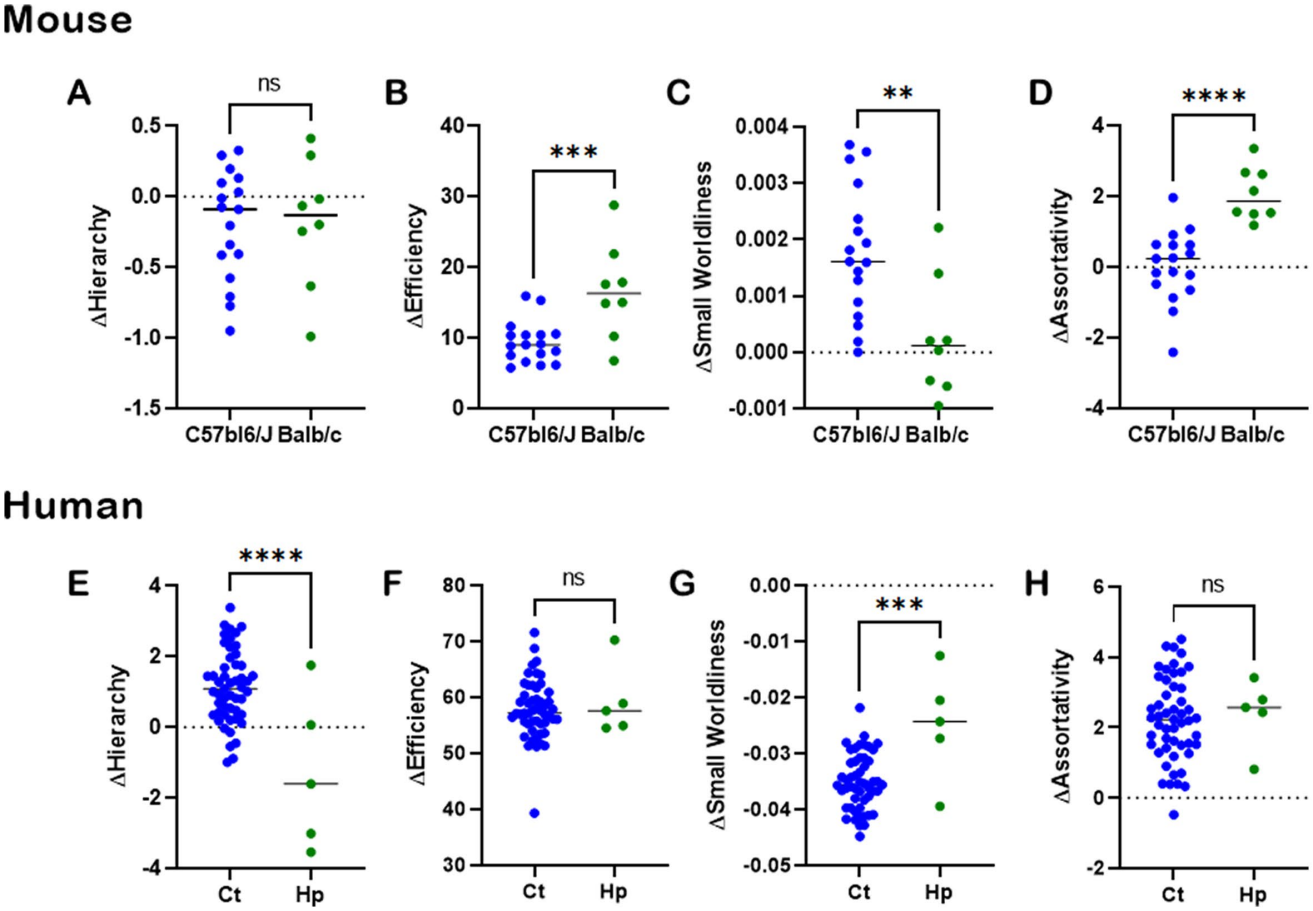
CCD impact to heterotopic connectivity. Analysis of CCD impact on heterotopic connections of whole-brain network-based-statistics in mouse **(A–D)** and human **(E–H)**. The network properties of Hierarchy, Efficiency, Small Worldliness, and Assortativity were computed for the full network NBS minus the heterotopic connections NBS for each subject. There was a clear impact of heterotopic connectivity to the CCD phenotype, showing the importance of such connections to understanding brain function. Ns, not significant; ^*^p < 0.05, ^**^p < 0.01, ^***^p < 0.001, and ^****^p < 0.0001, unpaired *t*-tests.

To gauge the relevance of heterotopic connections in CCD, we first subtracted the NBS values of NH from FN (Figure 8—see Methods) and compared results between normotypical C57bl6/J control mice and Balb/c CCD mice (Figures 8A–D), and between healthy subjects and hypoplasic CCD patients (Figures 8E–H). There is a clear difference between the mouse strains on ΔEfficiency, ΔSmall Worldliness, and ΔAssortativity (Figures 8B–D), revealing a higher impact of heterotopic connectivity to the CCD phenotype.

In humans, there is a difference between healthy subjects and CCD patients in ΔHierarchy (Figure 8E) and ΔSmall Worldliness (Figure 8G), indicating a higher effect of heterotopic connections to CCD. However, this was not observed for ΔEfficiency (Figure 8F) and ΔAssortativity (Figure 8H), indicating an equal effect of heterotopic connections in both healthy and CCD brains.

## Discussion

Our results show that the CCD brain is ~6%–8% more heterotopic than the healthy brain, both in mice and humans. Furthermore, the spatial heterotopicity pattern is highly variable across CCD individuals, with most subjects presenting areas almost entirely heterotopic and several cortical regions without measurable callosal communication. We also found that the callosal heterotopicity in CCD mice is spatially clustered at the genus or the callosal remnant, while hypoplasic humans showed no apparent callosal heterotopicity pattern. We used graph theory and network-based statistics to show that the heterotopic connections have an essential role in both healthy and CCD brains. Lastly, we showed that heterotopic connections affect the NBS properties differently in CCD brains vs. healthy controls. Taken together, our results show that heterotopic connections are substantially altered by CCD.

### Heterotopicity in CCD

Neuroscientists have studied brain development for decades, especially the formation of the corpus callosum (LaMantia and Rakic, 1994; Wahlsten et al., 2003; Donahoo and Richards, 2009). Historically, the corpus callosum was viewed as a primarily homotopic structure (Zhou et al., 2013; Fenlon and Richards, 2015; Suárez et al., 2018; Mancuso et al., 2019) with few heterotopic connections (Houzel et al., 2002; Marconi et al., 2003; Lanz et al., 2017). However, recent comprehensive studies investigating callosal connectivity concluded that most callosal connections are, in fact, heterotopic (Swanson et al., 2017; Szczupak et al., 2022), and that these connections have an essential role in defining the properties of brain networks (Szczupak et al., 2022). These newly discovered predominance of heterotopic connections in the healthy brain called into question their role in developmental disorders of the corpus callosum. It is well described in the literature that due to the CCD, new heterotopic bundles can be formed. The sigmoid bundle, a heterotopic frontal parieto-occipital connection (Tovar-Moll et al., 2006; Paul et al., 2007; Jakab et al., 2015;Edwards et al., 2020; Szczupak et al., 2020a), is the most iconical example. Whole-brain structural connectome studies have also demonstrated that heterotopic connections are prevalent in congenital CCD humans (Owen et al., 2013a,b; Jakab et al., 2015; Szczupak et al., 2021b) and mice (Edwards et al., 2020; Szczupak et al., 2020a).

Here, we focused our work on the Balb/c mouse, a spontaneous CCD model which produces a wide range of callosal phenotypes (Wahlsten et al., 1992; Ozaki and Wahlsten, 1993; Livy and Wahlsten, 1997; Szczupak et al., 2020a) and in a small cohort of hypoplasic human subjects. We restricted our work to subjects presenting at least a callosal remnant in order to map callosal heterotopicity, excluding agenesis patients without callosal connections, which have altered brain connections patterns (Tovar-Moll et al., 2014; Siffredi et al., 2019; Szczupak et al., 2021a) but the relevance of heterotopicity cannot be established. Our data shows that CCD globally increases heterotopicity in both species similarly. However, differently from healthy controls that show a cohesive heterotopicity index (Supplementary Table 1), the spatial pattern of heterotopicity varied greatly across subjects, with several regions almost entirely heterotopic and many regions that could not project across the midline, even in the presence of a hypoplasic CC. Such variability was expected, as CCD is one of the most variable conditions in its genetic makeup (Paul, 2011), midline anatomy (Schell-Apacik et al., 2008), and brain connectivity patterns (Owen et al., 2013b; Jakab et al., 2015; Szczupak et al., 2020b).

### Anatomical callosal placement of heterotopic connections in CCD

In our previous study, we showed in three different mammalian species that the heterotopic connections form the core of the CC, with the homotopic connections located along its periphery (Szczupak et al., 2022). Here, we show that CCD significantly alters the relative spatial arrangement of homotopic and heterotopic connections. In CCD human subjects, we found that the CC of each individual had heterotopicity cluster(s) located in different portions of the CC. On the other hand, the heterotopic connections in Balb/c mice were clearly displaced toward the anterior CC near the genus. This finding agrees with our previous report showing high variability in the anterior cortical connectivity patterns in this mouse strain (Szczupak et al., 2020a). A possible reason for a high cohesivity in Balb/c phenotype is that this is an inbred mouse strain (Wahlsten et al., 2003; Burket et al., 2009) with a relatively stable genetic makeup. The Balb/c mouse strain has been reported to develop a late fusion of the anterior hemisphere (Livy and Wahlsten, 1997) and altered fiber development in the anterior pole (Rayêe et al., 2021) relative to other normotypic strains. Our results reinforce the early CC development time-window importance to the heterotopic connection development in the Balb/c strain. Here, we speculate that the Balb/c anterior CC has a developmental disorder that leads to misregulated interhemispheric crossings. Future studies shall investigate the molecular mechanisms that lead to the anterior CC malformation in the Balb/c strain.

### Importance of heterotopic connections

To evaluate the importance of these connections to brain function, we used a standard graph network approach that has been previously applied to CCD (Owen et al., 2013b; Jakab et al., 2015; Edwards et al., 2020; Siffredi et al., 2020; Szczupak et al., 2020a, 2021b) and analyzed specific structural network properties (Assortativity, Hierarchy, Small-Worldliness, and Efficiency—see Methods). Heterotopic connections are fundamental to these network properties in typical brains (Szczupak et al., 2022). Interestingly, Balb/c mice did not show any differences in the Hierarchy and Small Worldliness. These results indicate that the heterotopic connections in Balb/c are equally connecting nodes of different hierarchical orders and not altering the network balance. Also, these connections have a different organization since the small world patterns are preserved despite removing heterotopic connections.

In hypoplasic human subjects, there is a clear pattern of the contribution of heterotopic connections to the NBS despite the high variance of the cohort. Nevertheless, similar to mice, there were no changes in the Hierarchy. It is worth noticing that Hierarchy is the most variable metric in all species and phenotypes. Therefore, the lack of significance in hypoplasic subjects could be due to the small sample size. Future studies shall observe larger cohorts to describe this interaction better.

### Relevance of heterotopic connections to CCD

It is well known that CCD can cause new abnormal heterotopic bundles such as the sigmoid bundle (Tovar-Moll et al., 2006; Paul et al., 2007; Kasprian et al., 2013; Edwards et al., 2020; Szczupak et al., 2020a), although there are reports of normal mice also presenting weaker connections with a similar trajectory (Szczupak et al., 2020a). Other whole-brain CCD connectome studies have also shown heterotopic connections spread around the cortex (Szczupak et al., 2020a, 2021b). Our analysis showed that CCD alters Efficiency, Small Worldliness, and Assortativity but not Hierarchy in mice. This interesting combination indicates a whole-brain reorganization in CCD mice that does not alter the Hierarchy between nodes. Instead, these streamlines connect nodes redundantly in a non-small-world motif, segregating nodes with a similar degree (Assortativity), essentially changing the brain architecture.

In hypoplasic subjects, the presence of CCD caused changes in Hierarchy and Small Worldliness, but not Efficiency and Assortativity. These results differ from CCD mice, indicating that heterotopic connections have different connectivity patterns that suggest distinct developmental trajectories in Balb/c mice and Humans. The long-range heterotopic connections in humans have a clear small world pattern decreasing the global Hierarchy. However, discussing whether these connections are adaptative, maladaptative, or mixed is still premature. Future studies targeting these connections might clarify the role of heterotopic connections to brain function in healthy and CCD brains.

## Data availability statement

The raw data supporting the conclusions of this article will be made available by the authors, without undue reservation.

## Ethics statement

The studies involving human participants were reviewed and approved by IDOR Internal Ethics Committee. Written informed consent to participate in this study was provided by the participants’ legal guardian/next of kin. The animal study was reviewed and approved by NIH-NINDS Ethics Committee.

## Author contributions

DS and AS designed experiments and analyses. FT-M provided human CCD data. DS, AS, FT-M, and RL wrote the manuscript and contributed to the manuscript revision. All authors contributed to the article and approved the submitted version.

## Funding

This work was supported by the National Institutes of Health, National Institute of Neurological Disorders and Stroke grant RF1NS117486, the PA Department of Health grant SAP# 4100083102, the Foundation of the State of Rio de Janeiro (FAPERJ), National Council for Scientific and Technological Development (CNPq), as well as by intramural grants from D’Or Institute for Research and Education (IDOR).

## Conflict of interest

The authors declare that the research was conducted in the absence of any commercial or financial relationships that could be construed as a potential conflict of interest.

## Publisher’s note

All claims expressed in this article are solely those of the authors and do not necessarily represent those of their affiliated organizations, or those of the publisher, the editors and the reviewers. Any product that may be evaluated in this article, or claim that may be made by its manufacturer, is not guaranteed or endorsed by the publisher.
